# Uptake and metabolism of sulphated steroids by the blood–brain barrier in the adult male rat

**DOI:** 10.1111/jnc.14117

**Published:** 2017-08-03

**Authors:** M. Zeeshan Qaiser, Diana E. M. Dolman, David J. Begley, N. Joan Abbott, Mihaela Cazacu‐Davidescu, Delia I. Corol, Jonathan P. Fry

**Affiliations:** ^1^ Blood–Brain Barrier Research Group Institute of Pharmaceutical Science Faculty of Life Sciences & Medicine King's College London London UK; ^2^ Department of Neuroscience Physiology and Pharmacology University College London Gower Street London UK; ^3^ Present address: Pharmidex 14 Hanover Street London W1S 1YH UK

**Keywords:** blood–brain barrier, dehydroepiandrosterone sulphate, neurosteroid, pregnenolone sulphate, steroid sulphatase

## Abstract

Little is known about the origin of the neuroactive steroids dehydroepiandrosterone sulphate (DHEAS) and pregnenolone sulphate (PregS) in the brain or of their subsequent metabolism. Using rat brain perfusion *in situ*, we have found ^3^H‐PregS to enter more rapidly than ^3^H‐DHEAS and both to undergo extensive (> 50%) desulphation within 0.5 min of uptake. Enzyme activity for the steroid sulphatase catalysing this deconjugation was enriched in the capillary fraction of the blood–brain barrier and its mRNA expressed in cultures of rat brain endothelial cells and astrocytes. Although permeability measurements suggested a net efflux, addition of the efflux inhibitors GF120918 and/or MK571 to the perfusate reduced rather than enhanced the uptake of ^3^H‐DHEAS and ^3^H‐PregS; a further reduction was seen upon the addition of unlabelled steroid sulphate, suggesting a saturable uptake transporter. Analysis of brain fractions after 0.5 min perfusion with the ^3^H‐steroid sulphates showed no further metabolism of PregS beyond the liberation of free steroid pregnenolone. By contrast, DHEAS underwent 17‐hydroxylation to form androstenediol in both the steroid sulphate and the free steroid fractions, with some additional formation of androstenedione in the latter. Our results indicate a gain of free steroid from circulating steroid sulphates as hormone precursors at the blood–brain barrier, with implications for ageing, neurogenesis, neuronal survival, learning and memory.

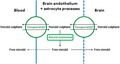

**Abbreviations used**
BBB: blood–brain barrier
BCRP: breast cancer‐resistance protein
cLogP_oct_: calculated logarithm of the octanol/water partition coefficient
DHEA(S): dehydroepiandrosterone (sulphate)
FS: free steroid
HLB: hydrophobic‐lipophilic balance
*K*_in_: unidirectional transfer co‐efficient
MAX: mixed‐mode anion exchange
MRP: multidrug resistance‐associated protein
OAT/oat: organic anion transporter
OATP/oatp: organic anion transporting polypeptide
P‐gp: P‐glycoprotein
Preg(S): pregnenolone (sulphate)
*PS*: permeability–surface area product
SG: steroid glucuronide
SS: steroid sulphate
*V*_d_: volume of distribution

Neuroactive steroids have a variety of actions within the central nervous system (CNS), and include the so‐called neurosteroids, synthesised within this tissue. The first proposed neurosteroid was dehydroepiandrosterone sulphate (DHEAS; Corpechot *et al*. 1981) but although enzymes are known which will catalyse the synthesis of this steroid (see Fig. 6), its origins in the CNS remain an enigma. Thus, early attempts to demonstrate activity in rat brain of the steroid 17α‐hydroxylase/c17,20‐lyase enzyme (EC 1.14.99.9; CYP17), which converts the precursor pregnenolone (Preg) to DHEA, were without success and this enzyme could not be detected by immunostaining of brain tissue sections (see Baulieu and Robel 1990; Le Goascogne *et al*. 1991). Indeed, further studies suggested the CYP17 mRNA and protein was only transiently expressed in rat brain during fetal development, although it persisted in adult dorsal root ganglia (Compagnone *et al*. 1995) and neither CYP17 mRNA nor enzyme activity could be detected in adult human brain (Steckelbroeck *et al*. 2004). On the other hand, low levels of CYP17 mRNA have been reported in adult rat (Stromstedt and Waterman 1995) and human (Yu *et al*. 2002) brain and both CYP17 mRNA and enzyme activity detected in astrocytes and neurons cultured from neonatal rat cerebral cortex (Zwain and Yen 1999). More recent studies have shown the expression of CYP17 mRNA, immunoreactivity and enzyme activity in adult rat spinal cord (Kibaly *et al*. 2005) and brain, where it is localised to the hippocampus (see Hojo *et al*. 2011). There is also the suggestion that DHEA may arise in brain through a CYP17 independent pathway involving the reduction of sterol hydroperoxides (Prasad *et al*. 1994), although it is not established how such a mechanism could operate normally *in vivo*. Notwithstanding the above ambiguities in the synthesis of DHEA within the CNS, the 3β‐hydroxysteroid sulphotransferase enzyme (EC 2.8.2.2) which would catalyse its sulphation to DHEAS displays only low or non‐detectable activity in adult rat (Rajkowski *et al*. 1997) or human (Steckelbroeck *et al*. 2004) brain. Also, treatment of adult mice with systemic doses of DHEA sufficient to cause a 30‐fold increase in the concentration of this steroid in the brain had no significant effect on the concentration of DHEAS (Young *et al*. 1991). Thus, sulphation of endogenous DHEA is thought unlikely to be the source of DHEAS within the brain.

The origin of DHEA and DHEAS in the brain remains an important question because the latter is the major circulating steroid in the adult primate, where it declines with age (Orentreich *et al*. 1984; Campbell 2006) and is thought to act as a precursor for biologically active hormone in a variety of peripheral tissues, a form of intracrinology (see Labrie *et al*. 1995; Reed *et al*. 2005; Starka *et al*. 2015). Moreover, both DHEAS and DHEA are known to have actions on the CNS. Thus DHEAS has been shown to act as an antagonist at the GABA_A_ receptor and an agonist at σ‐receptors (see Maninger *et al*. 2009; Starka *et al*. 2015), albeit at concentrations likely to exceed those found normally in the adult rat brain (Ebner *et al*. 2006). No specific receptor has been identified for DHEA, but animal studies have shown peripheral administration of this steroid or its likely precursor DHEAS to be neuroprotective against a variety of insults, including the stress hormone corticosterone (Karishma and Herbert 2002), and to promote neurogenesis and neuronal survival (see Maninger *et al*. 2009). Many animal studies have also shown administration of DHEA or DHEAS to enhance learning and memory (see Sujkovic *et al*. 2012) and although these effects have not consistently been repeated in human subjects (see Maninger *et al*. 2009; Starka *et al*. 2015), supplementation with DHEA(S) to ameliorate depression and improve cognitive function in elderly or demented subjects with lower DHEAS/cortisol ratios (Herbert 1998) remains a possibility (Grimley Evans *et al*. 2006; Samaras *et al*. 2013).

Early investigations of brain DHEAS uptake in monkeys (Knapstein *et al*. 1968) showed injection of this steroid in the carotid artery to give rise to more free steroid than sulphoconjugate in the cerebral cortex and later studies in humans found a significant correlation between the concentrations of free DHEA in CSF and DHEAS in serum (Kancheva *et al*. 2010). Working in rats, Kishimoto and Hoshi (1972) reported intracardiac injection of ^3^H‐DHEAS to result in nearly 50% of the radiolabel accumulating as free steroid in the brain, as compared to only 5% after intracerebral injection, suggesting desulphation before or during passage through the blood–brain barrier (BBB).

In view of the above, we hypothesised that DHEAS was taken‐up and metabolised at the BBB and tested this by direct perfusion of the adult rat brain with the steroid sulphate in a saline‐based solution *in situ*, in order to eliminate any complications of metabolism in blood or peripheral tissues. We employed male rats to avoid fluctuations in brain steroid concentrations during the ovarian cycle (Corpechot *et al*. 1997). For comparison, we also investigated the uptake and metabolism of the less well‐studied pregnenolone sulphate (PregS) which is also neuroactive, in particular as an antagonist of the GABA_A_ receptor, a potentiator of the NMDA receptor and an agonist at σ‐receptors (see Vallee *et al*. 2001; Monnet and Maurice 2006) and which on desulphation would give rise to pregnenolone, the precursor to all known steroid hormones (see Miller and Auchus 2011). Our results indicate rapid and extensive de‐esterification of steroid 3β‐sulphates at the BBB.

## Methods

### Materials

Chemicals of analytical grade were from Sigma‐Aldrich Chemical Co. (Poole, Dorset, UK) or VWR (Lutterworth, Leicestershire, UK). The former was the source of aryl sulfatase, Type H1 from *Helix pomatia* and some unlabelled reference steroid standards; others were from Steraloids Inc. (Newport, RI, USA). Ethanol, ethyl acetate and isooctane were redistilled before use. Of the radiolabelled chemicals, [^14^C]‐sucrose (22.8 GBq/mmol; 615 mCi/mmol) was from Amersham Biosciences (Little Chalfont, Buckinghamshire, UK) whilst [methyl‐^3^H]‐diazepam (3052.5 GBq/mmol; 82.5 Ci/mmol), [1,2,6,7‐^3^H]‐dehydroepiandrosterone (3422.5 GBq/mmol; 92.5 Ci/mmol) and [7‐^3^H]‐pregnenolone (777.0 GBq/mmol; 21 Ci/mmol) were from PerkinElmer LAS Ltd., Beaconsfield, Buckinghamshire, UK, as were Soluene, Solvable and HiSafe Optiphase 2. The Ecoscint H was from National Diagnostics, Hessle, Yorkshire, UK. The GF120918 (Elacridar; 9,10‐dihydro‐5‐methoxy‐9‐oxo‐N‐[4‐[2‐(1,2,3,4‐tetrahydro‐6,7‐dimethoxy‐2‐isoquinolinyl)ethyl]phenyl]‐4‐acridine‐carboxamide hydrochloride) was a gift from K Read (GlaxoSmithKline, Ware, Hertfordshire, UK) and MK571 (3‐[[[3‐[(1E)‐2‐(7‐Chloro‐2‐quinolinyl)ethenyl]phenyl][[3‐(dimethylamino)‐3‐oxopropyl]thio]methyl]thio]propanoic acid) from Alexis Corporation (UK) Ltd (Nottingham, UK). Hypnorm (fentanyl citrate 0.315 mg/mL and fluanisone 10 mg/mL) came from Janssen Animal Health (High Wycombe, Buckinghamshire, UK), Hypnovel (midazolam 5 mg/mL) from Roche Products (Welwyn Garden City, Hertfordshire, UK) and heparin from Leo Laboratories (Hurley, Berkshire UK). The Oasis Hydrophobic‐Lipophilic Balance (HLB^®^) and Mixed‐Mode Anion Exchange (MAX^®^) cartridges came from Waters Corporation, Milford, MA, USA and the Silica gel 60 coated thin layer chromatography (TLC) plates from VWR. The imaging film was BAS‐TR2040S from Raytek Scientific Ltd., Sheffield, South Yorkshire, UK. Silanised glassware was used throughout and unless stated otherwise, all samples dried down under oxygen‐free nitrogen.

### Sulphation of steroids

The ^3^H‐Preg and ^3^H‐DHEA were sulphated as described by Dusza *et al*. (1968) and then cleaned by procedures given in Corsan *et al*. (1997). In brief, ^3^H‐steroids were dried down and redissolved in 10 μL chloroform. Solid triethylamine sulphur trioxide was added in excess and the reaction mixture left for 2 h at 45°C. For cleaning, reaction mixtures were dried down, redissolved in 2 mL of 5 mM sodium phosphate buffer, pH 7 and washed three times with an equal volume of isooctane to remove remaining free steroid. Solid sodium chloride was then added to 20% w/v and the steroid sulphates extracted three times with an equal volume of ethyl acetate. Finally, the steroid sulphates were purified by celite chromatography in isooctane:tert‐butanol:water:ammonia (20 : 40 : 39 : 1, v/v). The radioactive fractions were pooled, dried down under vacuum and redissolved in ammoniated ethanol. Yields of the initial steroid sulphation reactions and purity of the final products could be estimated by TLC in ethyl acetate:ethanol:ammonia (25 : 10 : 2, v/v). Positions of the ^3^H‐steroids and their sulphate esters were visualised with a phosphorimager and in both systems corresponded to non‐radioactive standards (50 μg) run on neighbouring lanes and stained with I_2_ vapour. Measurement of the relative densities of the spots corresponding to the ^3^H‐steroids and their sulphate esters gave estimates for the yields of the initial sulphation reaction > 94% and for the purity of the final product > 99% (see Fig. S1).

### Brain perfusion *in situ*

All animal experiments were carried out under Home Office Project Licence PPL/5224 according to the UK Animals (Scientific Procedures) Act 1986, The European Directive for the Protection of Animals used in Scientific Procedures 2008 and the Arrive Guidelines (www.nc3rs.org.uk/arrive-guidelines). Brain perfusions were performed using a modified short‐duration technique (Takasato *et al*. 1984). Briefly, male Wistar rats purchased from Harlan (now Envigo; Resource Identifier RRID:RGD_10401918), Bicester, UK (age 6–8 weeks; body weight range 225–365 g) were anaesthetised by an intra‐peritoneal injection (2.7 mL/kg) of water:Hypnorm:Hypnovel, 2 : 1 : 1, v/v. Each animal also received intraperitoneal heparin, 10 000 U/kg. Body temperature was maintained at 37°C with a heating pad.

Hemi‐perfusions of the right hemisphere were carried out via the right common carotid artery (Youdim *et al*. 2004). Perfusions were performed using a pre‐oxygenated (95% O_2_, 5% CO_2_), filtered and saline‐based solution containing (in mM) 117 NaCl, 4.7 KCl, 24.8 NaHCO_3_, 1.2 KH_2_PO_4_, 2.5 CaCl_2_, 0.8 MgSO_4_, 10 HEPES and 10 D‐glucose, pH 7.4, at 37°C. For analysis of free and sulphated steroids and for steryl‐sulfate sulfohydrolase (EC 3.1.6.2; trivial name steroid sulphatase) assays, both hemispheres were perfused, via right and left common carotid arteries, and 0.8 mM MgCl_2_ replaced 0.8 mM MgSO_4_ in the solution. The perfusate was delivered at a constant rate of 6 mL/min for hemi‐perfusions and 12 mL/min for bilateral perfusions, maintaining a net perfusion pressure of 40***–***70 mm Hg.

### Sample collection and analysis

On completion of the perfusion, the animal was decapitated for rapid removal of the brain. The meninges and choroid plexus were stripped away, the hindbrain cut off and the remaining brain divided into right and left hemispheres. The perfusion and subsequent processing of the brain samples followed one of three procedures given below.

#### 1. Uptake of ^3^H‐steroid

The saline‐based solution containing 0.5 μCi (0.0185 MBq)/mL ^3^H‐labelled DHEAS (5.4 nM) or PregS (23.8 nM) was used for hemi‐perfusions of up to 1.0 min. Influence of efflux transporters on uptake was assessed by adding 3 μM GF120918 and/or 50 μM MK571 directly to the perfusate, and co‐perfusing them with the test compound, as in the protocol of (Cisternino *et al*. 2003a). In additional perfusions, saline containing the impermeant marker ^14^C‐sucrose (0.1 μCi (0.0037 MBq)/mL; 162.6 nM) was used to determine the intravascular space, or saline containing ^3^H‐diazepam (1.0 μCi (0.037 MBq)/mL; 12.1 nM), which shows flow‐limited uptake, to measure the flow rate of cerebral perfusate.

Samples (≤ 50 mg) were taken from 7 regions of the right brain (cerebellum, cortex, hippocampus, hypothalamus, striatum, superior colliculus, and medulla). The remaining tissue of the right hemisphere was homogenised and samples (~50 mg) taken in triplicate (“remainder samples”). Triplicate samples of the perfusate were also taken. Samples were weighed into vials and dissolved (48 h at 20°C) in 0.5 mL Solvable before addition of 4.5 mL Optiphase 2 scintillation fluid for counting of radioactivity.

#### 2. Extraction and separation of free and sulphated ^3^H‐steroids

Brains were bilaterally perfused for 0.5 min with ^3^H‐labelled DHEAS 1.25 or 6.0 μCi (0.046 or 0.221 MBq)/mL (13.5 or 64.9 nM) or PregS 1.0 μCi (0.037 MBq)/mL (47.6 nM). The hindbrain was removed and each hemisphere homogenised in 2 vols (w/v) buffer containing (in mM) NaCl 141, KCl 4, NaH_2_PO_4_ 1, CaCl_2_ 2.8, MgCl_2_ 1, HEPES 10, D‐glucose 10, pH 7.4. A sample of the homogenate of the whole right hemisphere was removed for scintillation counting, and the remainder retained as the whole brain homogenate.

The left hemisphere homogenate was separated into capillary‐enriched and capillary‐depleted fractions by the method of Triguero *et al*. (1990). For this, an equal volume of 26% dextran (w/w) solution was added to the homogenate and a sample taken for liquid scintillation counting before centrifugation at 5300 *g* for 15 min at 4°C. The upper layer was taken as the capillary‐depleted fraction and the pellet as the capillary‐enriched fraction.

Before extraction, fractions were sonicated in 5 vols ice‐cold, 5 mM potassium phosphate buffer, pH 7. Small portions (150 μL) were removed for measurement of protein content (Bradford 1976) and total radioactivity. For the latter, homogenate was solubilised (15 min, 20°C) in 10 vols Soluene then bleached (2 h, 50°C) with 0.3 vol hydrogen peroxide before counting in Ecoscint H containing Triton X‐100 (6% v/v), glacial acetic acid (0.6% v/v) and butylated hydroxytoluene (2% w/v). The remainder of each homogenate was extracted as described in detail elsewhere (Ebner *et al*. 2006; Sujkovic *et al*. 2009), to give free steroid and steroid sulphate fractions. First, homogenates were extracted into 20 vols ethanol containing acetic acid 3% (v/v). After centrifugation (28 000 *g*, 30 min, 25°C) to remove denatured protein, the extracts were delipidated by partitioning three times against 10 vols isooctane. Extracts were dried down under vacuum and redissolved in ethanol, which was then diluted to 20% v/v in 5 mM potassium phosphate buffer, pH 7.0 at a total volume of 20 ml. Separation of free and sulphated steroids was achieved by passage through a 60 mg Oasis MAX^®^ cartridge. After washing the cartridge with 5 mL ethanol at 20% v/v in ammonium acetate (20 mM, pH 7), free steroids were eluted in 4 mL ethyl acetate. Passage of 20 mL ethanol at 60% v/v in formate/pyridine buffer (20 mM, pH 3) eluted any steroid glucuronides present. Finally, steroid sulphates were eluted in 30 mL 60% v/v ethanol containing ammonium carbonate 1% (w/v). To measure the radioactivity of each fraction, portions were dried down and then redissolved in Ecoscint H for counting. The remainder was dried down, redissolved in ethanol 96% v/v and stored at −20°C for subsequent analysis by TLC.

For the analysis of ^3^H‐steroid metabolites, samples were spotted onto TLC plates for chromatography in the following solvent systems: A (for sulphated steroids), ethyl acetate:ethanol:ammonia (5 : 5 : 1, v/v); B (for free steroids) cyclohexane:n‐butyl acetate (1 : 2, v/v); C (for acetylated steroids), isooctane:ethyl acetate (13 : 7, v/v). Non‐radioactive steroid standards could be visualised by exposure to iodine vapour whereas the ^3^H‐steroids were detected by placing the TLC plates in contact with imaging film for subsequent visualisation in a phosphorimager. Additional analysis of the steroid sulphates employed elution from the TLC plate in ammoniated ethanol then drying down and redissolving in sodium acetate buffer, 0.5 M, pH 5.0 for desulphation by incubation with *Helix* sulphatase (1 mg/mL, 40°C overnight followed by 55°C for 3 h). The resulting free steroids were adsorbed onto a 60 mg Oasis HLB^®^ cartridge and eluted in 5 mL ethyl acetate. This eluate was dried down and analysed by TLC as above for free steroids. Further identification of ^3^H‐steroid metabolites was obtained by acetylating them alongside both ^3^H‐labelled and non‐radioactive standards. The TLC eluates or standards were dried down and redissolved in 200 μL pyridine. An equal volume of acetic anhydride was then added followed by incubation at 37°C for 2 h. The reaction was stopped by the addition of 1 mL water and the steroids extracted three times with 1 mL ethyl acetate before drying down for TLC and visualisation of both ^3^H‐labelled and non‐radioactive standards as described above.

#### 3. Assay of steroid sulphatase

Brains were bilaterally perfused for 2 min. The hindbrain was removed and the right hemisphere homogenised as the whole brain homogenate sample, whilst the left hemisphere was separated into capillary‐enriched and capillary‐depleted fractions, as above.

Before assay, fractions were sonicated on ice in 12 vols 50 mM potassium phosphate buffer, pH 7.0, then incubated at 200 μg protein per assay tube with ^3^H‐DHEAS at 2 nM in the same buffer in a total volume of 250 μL. In addition to buffer blanks, boiled blanks (3 min, 100°C) were included for each sample and all assayed in duplicate. After 3 h at 37°C, incubations were terminated by the addition of 750 μL 0.1 M NaOH. Free ^3^H‐DHEA was then extracted twice into 2.0 mL toluene containing non‐radioactive DHEA at 25 μg/mL. This organic phase was dried down for spotting onto TLC plates, which were developed in solvent system B. Exposure of the plates to I_2_ vapour allowed visualisation of the DHEA spots, which were cut out and placed in scintillation vials with Ecoscint H for measurement of radioactivity. Production of DHEA from DHEAS by sulphatase was estimated by subtraction of the boiled blank for each tissue sample.

## Reverse‐transcriptase–polymerase chain reaction

Samples were obtained from regions of adult male rat brains and primary cultures of rat brain microvessel endothelial cells and astrocytes for extraction of RNA, RT–PCR and cloning as described in Dolman *et al*. (2005). At least three independent samples were tested. The internal control was β‐actin. Reverse transcriptase was omitted from negative controls. Amplification employed 25 cycles for actin and 35 cycles for steroid sulphatase. The PCR products were separated on 1% agarose gels stained with ethidium bromide, and visualised with ultraviolet illumination. Sequences of the primers for steroid sulphatase (accession number NM012661) were 860–879: CCCAATGAAGTCACCTTTGC; and 1288–1307: GTGTGAGCCCCTTGTAGTCC; expected product size 448 bp. For β‐actin (accession number V01217) they were 1343–1363: ATCGTGGGCCGCCCTAGGCAC and 1652–1673: TGGCCTTAGGGTTCAGAGGGGC; expected product size 243 bp.

## Analysis of results

Brain uptake was calculated as a volume of distribution *V*_d_, where *V*_d_ (μL/g) = (dpm/g tissue)/(dpm/μL perfusate). The unidirectional transfer coefficients, (*K*_in_, μL/g/min), for DHEAS and for PregS were calculated from the *V*_d_ at 0.5 min, corrected with the *V*_d_ for the vascular space marker sucrose. Values for *K*_in_ were converted into the permeability–surface area *PS* product (μL/g/min) using the Renkin–Crone equation (Renkin 1959; Crone 1963): *PS* = −*vF* ln (1−*K*_in_/*vF*), where *P* is the capillary permeability (cm/min), *S* is the surface area of perfused capillaries (cm^2^/g), *v* is the fractional distribution volume of the tracer in red blood cells (mL/mL), and *F* is the cerebral blood flow (mL/g/min). As a saline‐based perfusate was used for the present experiments, *vF* can be replaced by *F,* the *K*_in_ value for the flow‐limited marker diazepam, estimated from a linear least squares fit of a plot of *V*_d_ against time.

The values for the calculated logarithm of the octanol/water partition coefficient, Log P_oct_ (cLogP_oct_), were obtained from LogKow software (http://www.epa.gov/oppt/exposure/pubs/episuite.htm ).

Film for imaging ^3^H‐activity on TLC plates was visualised in a Typhoon 9410 Variable Mode phosphorimager for analysis with ImageQuant software (Molecular Dynamics, Amersham, Buckinghamshire, UK).

## Statistics

Linear regression lines with confidence limits were fitted in GraphPad Prism and statistical significance of results evaluated with Student's *t* test or anova, with *p *<* *0.05 accepted as significant and the sample sizes judged to give > 80% power (http://www.3rs-reduction.co.uk). Rats were allocated at random to treatments of which the investigators were aware.

## Results

### PregS enters the brain more rapidly than DHEAS

**Figure 1:**
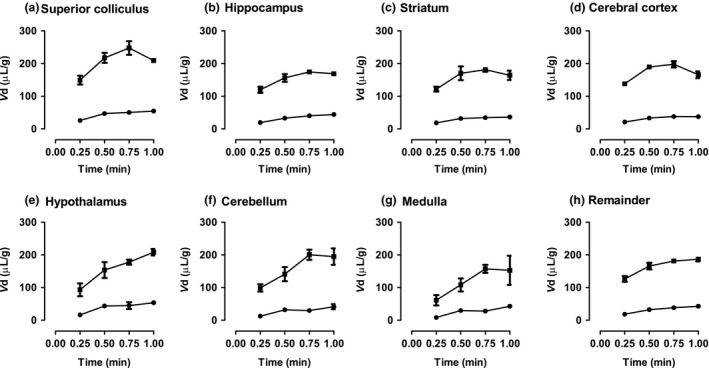
Plot of volume of distribution (V_d_) against time for uptake of ^3^H‐dehydroepiandrosterone sulphate (DHEAS; ●) and ^3^H‐pregnenolone sulphate (PregS; ■) into (a) to (g) different regions of the brain plus (h) the Remainder (mean ± SEM, n ≥ 3 animals at each time point; error bars smaller than symbols for DHEAS).

Uptake (distribution volume, *V*_d,_ in μL/g) was measured at four time‐points, as shown in Fig. 1 for the different brain regions. For all regions, the initial entry of PregS into the brain was greater than that of DHEAS and reached a maximum within 1 min.

### When adjusted for perfusate flow rate, different brain regions show similar permeabilities for steroid sulphates

The regional uptake of DHEAS and PregS after correction for vascular space is shown in Table 1 as the unidirectional transfer coefficients at 0.5 min, *K*_in_ (μL/g/min). The only significant differences found in *K*_in_ were for PregS between the superior colliculus and medulla and for DHEAS between the superior colliculus and the cerebellum. However, after adjustment for perfusate flow rate (using the *K*_in_ for diazepam) and conversion to the permeability–surface area product (*PS*), there was no significant difference in *PS* value for either sulphated steroid between the brain regions analysed.

**Table 1:** Regional unidirectional transfer coefficients, *K*_in_, and permeability (permeability–surface area product, *PS*) for dehydroepiandrosterone sulphate (DHEAS) and pregnenolone sulphate (PregS) after 0.5 min perfusion (all in μL/g/min)

Brain region	*V*_d_ Sucrose	*K*_in_ Diazepam	*K*_in_ DHEAS	*K*_in_ PregS	*PS* DHEAS	*PS* PregS
Sup. colliculus	15.0 ± 0.9	5898.2 ± 1118.4	63.5 ± 6.7†	404.9 ± 30.4*	63.8 ± 6.8	419.6 ± 32.5
Hippocampus	9.9 ± 1.5	4028.8 ± 818.7	45.4 ± 3.4	292.1 ± 24.2	45.6 ± 3.4	303.5 ± 26.0
Striatum	5.6 ± 0.1	2694.6 ± 791.7	47.2 ± 3.9	328.6 ± 41.9	47.6 ± 4.0	351.5 ± 48.1
Hypothalamus	14.6 ± 0.7	2461.2 ± 631.2	57.7 ± 2.7	277.6 ± 48.8	58.4 ± 2.7	295.8 ± 54.8
Parietal cortex	13.6 ± 1.4	1617.6 ± 772.3	38.4 ± 4.5	351.5 ± 9.8	38.7 ± 4.6	377.2 ± 11.3
Cerebellum	12.5 ± 1.4	471.5 ± 193.4	38.6 ± 7.0†	256.8 ± 43.7	40.4 ± 7.6	392.6 ± 103.4
Medulla	9.8 ± 0.3	339.7 ± 147.9	39.4 ± 8.9	196.4 ± 39.5*	42.4 ± 10.0	328.6 ± 117.5
Remainder	10.1 ± 0.8	3363.3 ± 446.2	44.8 ± 4.1	311.2 ± 20.8	45.2 ± 4.1	326.5 ± 22.9

The *K*_in_ measurements for DHEAS and PregS have been corrected for vascular space as measured by the *V*_d_ for sucrose (in μL/g). Conversion of these *K*_in_ measurements to *PS* values by the Renkin–Crone equation (see Methods) used the *K*_in_ for the flow marker diazepam, as shown. All values mean ± SEM; *n* ≥ 3. ‘Remainder’ is the brain tissue remaining after the removal of regional samples. *p *<* *0.05 for significance of difference between brain regions after anova as indicated by * and †.

### Analysis of sulphated steroid BBB permeability

**Figure 2:**
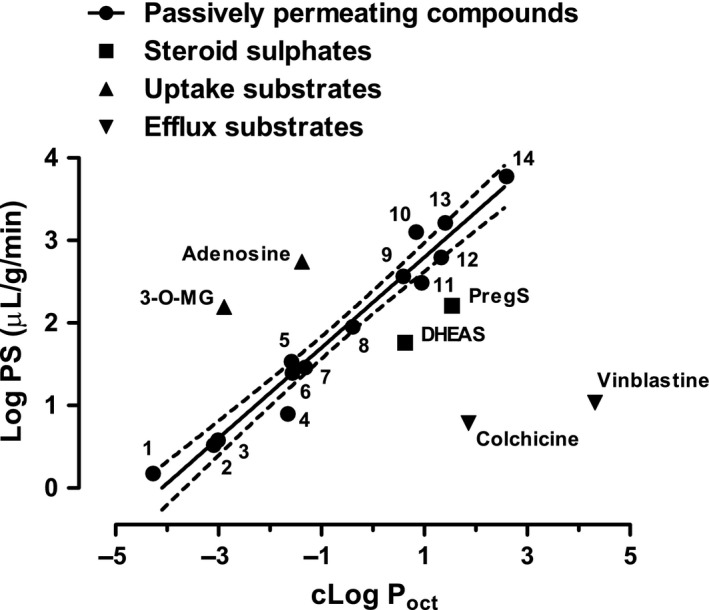
Plot of permeability (Log *PS*) against calculated lipophilicity (cLog P_oct_) for compounds known to enter the brain passively, compounds known to be substrates for active uptake or efflux, and the two sulphated steroids dehydroepiandrosterone sulphate (DHEAS) and pregnenolone sulphate (PregS), *n **≥** *3 rats for each compound. Passively permeating compounds are numbered in order of increasing calculated lipophilicity: 1, Sucrose; 2, Inulin; 3, Mannitol; 4, Glycerol; 5, Pyridostigmine; 6, Urea; 7, Thiourea; 8, Theophylline; 9, Antipyrine; 10, Butanol; 11, Naloxone; 12, Physostigmine; 13, Iodo‐Antipyrine; 14, DL‐Propanolol. For these passively permeating compounds there is a significant correlation with calculated lipophilicity (*p *<* *0.0001; Spearman coefficient = 0.9648). The linear regression line is drawn through these numbered points, with a slope of 0.5480 and with 95% confidence limits shown by the dotted lines. Permeability measurements from Youdim *et al*. (2004) and from (Qaiser 2004).

The *PS* values for PregS and DHEAS were compared with those for compounds known either to enter the brain passively or to be substrates for influx or efflux transporters (Qaiser 2004; Youdim *et al*. 2004). Log *PS* was plotted against cLog *P*_oct_, an index of lipophilicity (see Fig. 2). For the passively permeating substances, there is a linear relation between permeability and lipophilicity. Compounds known to be substrates for uptake transport lie above and substrates for efflux below this line. For consistency with the method used for the reference data set, *PS* values for DHEAS and PregS were calculated from *K*_in_ values obtained for the slope of a plot of *V*_d_ against time from 0.25 to 0.5 min. The log *PS* values for both PregS and DHEAS appear below the 95% confidence limits of the line of best fit for passively permeating compounds, implying net efflux, but not as far below as the known efflux transport substrates colchicine and vinblastine. We therefore tested the effect of known efflux inhibitors on the brain uptake of PregS and DHEAS.

**Figure 3:**
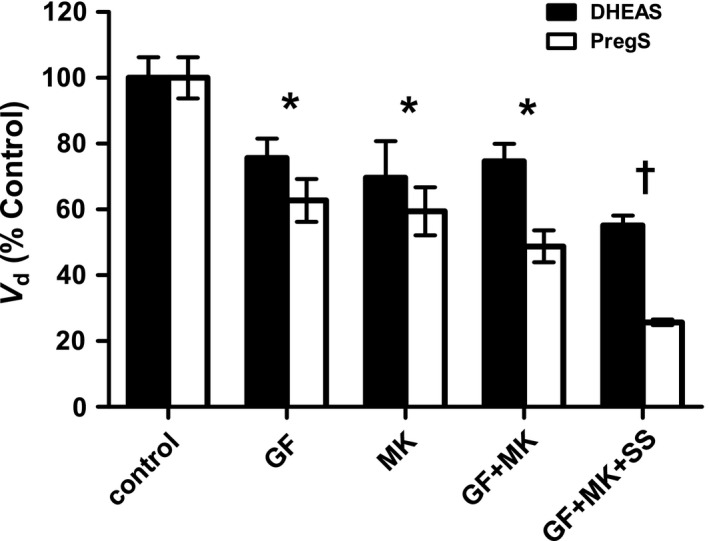
Effect of the efflux inhibitors GF120918 (3 μM) and MK571 (50 μM), alone or in combination, on the brain uptake of either ^3^H‐dehydroepiandrosterone sulphate (DHEAS) or ^3^H‐pregnenolone sulphate (PregS) after 0.5 min perfusion. The *V*_d_, corrected for sucrose space (vascular volume), is shown as % control, 32.47 ± 2.03 μL/g for DHEAS and 165.63 ± 10.40 μL/g for PregS. All values mean ± SEM,* n *=* *3. The effect of 0.5 mM of the corresponding unlabelled steroid sulphate (SS) in addition to the inhibitors is also shown. The same *p *<* *0.05 values (anova, with Dunnett's *post hoc* test) are indicated for both DHEAS and PregS; * versus control and † versus GF120918 plus MK571 alone. The inhibitors GF120918 and MK571 were dissolved in dimethylsulphoxide to a maximum final concentration of 0.8% (v/v) in the perfusate. Control experiments showed that this vehicle alone did not cause a change in the uptake (data not shown).

When the efflux transporter inhibitors GF120918 (3 μM; Polli *et al*. 2004) or MK571 (50 μM; Cisternino *et al*. 2003b), alone or in combination, were co‐perfused with either ^3^H‐PregS or ^3^H‐DHEAS there was a significant decrease in uptake (Fig. 3). Moreover, addition of the corresponding unlabelled steroid sulphate (0.5 mM) caused a further decrease in the uptake of ^3^H‐steroid sulphate.

### PregS and DHEAS are rapidly desulphated upon uptake into the brain

**Figure 4:**
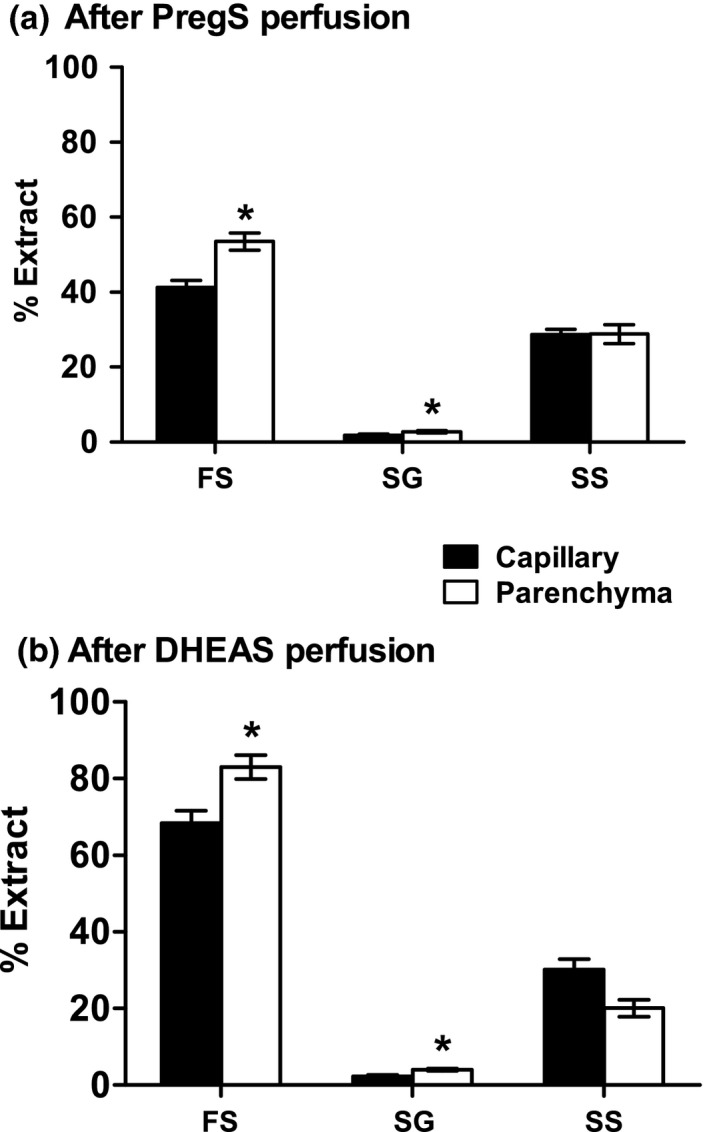
Percentage recoveries of free steroids (FS), steroid glucuronides (SG) and steroid sulphates (SS) from capillary and parenchyma following brain perfusion with (a) ^3^H‐pregnenolone sulphate (PregS) or (b) ^3^H‐dehydroepiandrosterone sulphate (DHEAS). All values mean ± SEM;* n *=* *5. For significance of difference between capillary and parenchyma: *p <* *0.02. Control experiments in which standard ^3^H‐DHEAS, ^3^H‐DHEA, ^3^H‐PregS or ^3^H‐Preg were added to rat brain homogenates followed by extraction and separation as for the samples above showed recovery into free steroid (FS), steroid glucuronide (SG) and steroid sulphate fractions as follows. 1). ^3^H‐DHEAS: FS 1.89 ± 0.28%; SG 0.18 ± 0.01%; SS 74.15 ± 0.92%. 2). ^3^H‐DHEA: FS 94.62 ± 0.70%; SG 2.55 ± 0.08%; SS 0.18 ± 0.01%. 3). ^3^H‐PregS: FS 6.84 ± 0.69%; SG 0.13 ± 0.02%; SS 42.03 ± 1.29%. 4). ^3^H‐Preg: FS 102.01 ± 4.00%; SG 2.26 ± 0.24%; SS 0.25 ± 0.03%. Further TLC analysis of these fractions is described under Supporting Information.

After perfusion with either ^3^H‐PregS or ^3^H‐DHEAS, brains were homogenised and fractionated into capillary‐enriched (capillary) and capillary‐depleted (parenchyma) fractions. The % recovery of total homogenate radioactivity into these fractions was as follows (mean ± SEM, *n *=* *5 and also shown as dpm/mg protein): after PregS perfusion 8.8 ± 1.8% (1856 ± 245 dpm/mg protein) capillary and 68.0 ± 10.0% (1988 ± 328 dpm/mg protein) parenchyma; after DHEAS perfusion 2.2 ± 0.3% capillary (3451 ± 511 dpm/mg protein) and 25.7 ± 2.3% parenchyma (3279 ± 388 dpm/mg protein). These homogenate fractions were then extracted prior to separation of free and conjugated steroids. Extraction efficiencies for total radioactivity from these fractions were as follows: after PregS perfusion 76.0 ± 2.3% from capillary; 88.7 ± 4.3% from parenchyma and after DHEAS perfusion 80.0 ± 1.3% from capillary; 98.6 ± 8.2% from parenchyma. Finally, the extracts were separated into free steroid (FS), steroid glucuronide (SG) and steroid sulphate (SS) fractions by passage through mixed‐mode anion exchange cartridges. Figure 4 shows the percentage of ^3^H‐label recovered in each fraction. After perfusion with either ^3^H‐PregS or ^3^H‐DHEAS, a high proportion of the activity was recovered in the FS fraction, and for both labels, there was a significantly higher percentage of the free steroid in the parenchyma than in the brain capillaries. Control experiments (*n *=* *4) in which standard ^3^H‐DHEAS or ^3^H‐PregS were added to rat brain homogenates followed by extraction and separation into FS, SG and SS fractions, confirmed that their rapid desulphation seen above on brain uptake occurred *in vivo* and was not an artefact of the extraction and fractionation procedure (see Fig. 4 legend). As expected, negligible activity was recovered as SG and could be accounted for by spill over from the FS fraction.

### After uptake into the brain, DHEAS but not PregS is rapidly metabolised in both the sulphated and desulphated form

Identities of the steroids present in the FS and SS fractions from the parenchyma (*n *=* *5) of either ^3^H‐PregS‐ or ^3^H‐DHEAS‐perfused rat brains were investigated by TLC alongside both ^3^H‐labelled and non‐radioactive standards (*n *≥* *2), the former visualised in a phosphorimager and the latter by iodine staining. Positions of these steroids on TLC are given under Supporting Information (and illustrated in Figs S2–S6).

Upon TLC, the SS fractions from both ^3^H‐PregS‐ and ^3^H‐DHEAS‐perfused rat brains gave single peaks corresponding to their appropriate standards. Identity of these sulphated steroids was investigated by eluting the peaks and desulphating the ^3^H‐label for further TLC as free steroids alongside standard compounds. Confirmation of the identity of these free steroids could then be sought by eluting them for acetylation together with known standards before additional TLC.

Following desulphation and further TLC, the ^3^H‐label from the SS fractions of ^3^H‐PregS‐perfused rat brains gave a single peak corresponding with both the desulphated ^3^H‐PregS and the free Preg standards. Likewise, TLC of the FS fraction from these ^3^H‐PregS‐perfused rat brains in the same system showed no evidence of metabolism other than desulphation, with peaks corresponding to standard Preg and not to other possible Preg metabolites. These putative Preg peaks from the desulphated material of the SS fraction and from the FS fractions were eluted for acetylation alongside both ^3^H‐labelled and non‐radioactive standard Preg and other steroids. On subsequent TLC, both standard ^3^H‐Preg and standard desulphated ^3^H‐PregS gave two peaks, corresponding with those from the acetylated putative Preg peaks of the FS fractions and of the desulphated SS fractions. These two peaks arise from a presumed Serini reaction in which the Preg forms isomeric enol acetates (Fieser and Huang‐Minlon 1949).

From the ^3^H‐DHEAS‐perfused rats, there was evidence of metabolism in both the desulphated SS and the FS fractions, which each gave two peaks. The earlier peaks from both fractions corresponded with androstenediol and the later peaks with ^3^H‐labelled and non‐radioactive DHEA. There were no detectable 7‐hydroxymetabolites of DHEA. The two peaks which arose from the SS fraction were not an artefact of the deconjugation procedure because desulphation of the ^3^H‐DHEAS standard gave only one peak. Identity of the two peaks from the SS and the FS fractions was confirmed by eluting them for acetylation alongside standards for further TLC. On acetylation, the putative DHEA from both the SS and the FS fractions corresponded with acetylated standard ^3^H‐labelled DHEA and non‐radioactive DHEA. Acetylation of the putative androstenediol gave peaks from the SS fractions and from the FS fractions which corresponded with acetylated standard androstenediol. However, acetylation of the ^3^H‐label eluted from the putative androstenediol peak in the FS fraction also gave two additional peaks, one of which corresponded to standard androstenedione carried through the acetylation procedure (although this would not be acetylated) and another which could not be identified. There was no ^3^H‐peak corresponding with standard acetylated testosterone.

Control experiments (*n *=* *4) in which standard ^3^H‐DHEAS, ^3^H‐DHEA, ^3^H‐PregS or ^3^H‐Preg were added to rat brain homogenates followed by extraction and separation into FS and SS fractions, as for the perfused rat brain samples, then subjected to TLC, showed these labels to be unchanged by the extraction and separation procedure (see Supporting Information).

### Steroid sulphatase activity is enriched in brain capillaries

**Figure 5:**
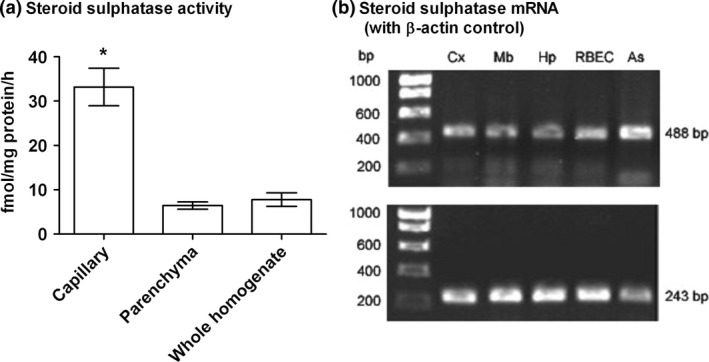
Steroid sulphatase activity is enriched in the blood–brain barrier and its mRNA expressed in rat brain. (a) Steroid sulphatase activity assayed with ^3^H‐dehydroepiandrosterone sulphate as the substrate in fractions of capillary, parenchyma and whole homogenate of rat brain. All values mean ± SEM,* n *=* *6; *p <* *0.001 in comparison to parenchyma. (b) Expression of mRNA for steroid sulphatase (upper pane) and β‐actin (lower pane) in rat brain samples; the expected band sizes in base pairs (bp) for PCR products are as shown: Cx, cortex; Mb, midbrain; Hp, hippocampus; RBEC, cultured rat brain microvessel endothelial cells; As, cultured rat astrocytes.

In view of the rapid desulphation of both ^3^H‐DHEAS and ^3^H‐PregS following uptake from the brain perfusate and the higher proportion of free steroid found in the brain parenchyma compared to the capillary, we assayed these two fractions of brain for sulphatase activity. Using ^3^H‐DHEAS as the substrate, sulphatase activity was found to be 5 times higher in the brain capillary than the brain parenchyma (see Fig. 5a).

### Steroid sulphatase mRNA is expressed in brain

Steroid sulphatase mRNA expression was investigated in brain samples and in cultured rat BBB‐related cells. Bands of the expected size for the steroid sulphatase PCR product were seen in all the brain regions investigated and in the samples of primary cultures of brain microvessel endothelial cells and astrocytes (Fig. 5b). Intensity of bands for β‐actin was similar in all samples. The identity of the products was confirmed by cloning. There were no bands seen in the negative controls (results not shown), showing that genomic DNA was not present.

## Discussion

Using direct perfusion with a saline‐based solution *in situ,* the present study has shown both ^3^H‐labelled steroid sulphates, PregS and DHEAS, to be taken up and metabolised by adult male rat brain. We perfused low concentrations of the steroid sulphates in a saline‐based solution to avoid protein binding and showed uptake of PregS to be more rapid than for DHEAS, presumably because of its higher lipophilicity. A large proportion (> 50%) of both labels was desulphated on uptake, but only DHEA and DHEAS underwent further metabolism during the 0.5 min perfusion of our experiments. Further investigation showed the steroid sulphatase responsible for deconjugating PregS and DHEAS to be enriched in the capillaries of the BBB, and the mRNA for this enzyme to be expressed in primary cultures of rat brain microvessel endothelial cells and astrocytes. Conventional light microscope immunohistochemical investigation of steroid sulphatase has revealed cytoplasmic staining in large neurones of the human cerebral cortex (Steckelbroeck *et al*. 2004). Higher resolution techniques will now be necessary to investigate the distribution of this enzyme in the smaller cells of the BBB.

One previous study (Wang *et al*. 1997b) showed differences in PregS concentrations between rat brain regions following intravenous infusion of this steroid. However, our results after carotid perfusion show no significant difference in regional permeability for either PregS or DHEAS after adjustment of regional uptake for perfusate flow. These measurements of perfusate flow confirm adequate and physiological perfusion of the brain regions examined and indicate that with a saline‐based perfusion fluid less viscous than blood, adequate perfusion can be maintained at pressures (40–70 mm Hg) close to the critical cerebral perfusion pressure of 50 mm Hg measured in the rat by Bragin *et al*. (2014).

Our analysis of BBB permeability suggested a net efflux of these steroid sulphates at the rat BBB, although not as much with respect to lipophilicity as the known substrates for efflux, colchicine and vinblastine. A net efflux of steroid sulphate from rat brain had been reported by Asaba *et al*. (2000) for DHEAS and Hosoya *et al*. (2000) for estrone sulphate. Thus, we did not expect the observed decrease in the uptake of PregS or of DHEAS upon co‐perfusion with the efflux transporter inhibitors GF120918 and/or MK571. The compound GF120918 inhibits P‐gp (P‐glycoprotein; ABCB1; ATP‐binding cassette transporter B1) and BCRP (breast cancer resistance protein; ABCG2) (Jonker *et al*. 2000) whilst MK571 inhibits MRPs (multidrug resistance associated proteins; ABCCs) (Gekeler *et al*. 1995). DHEAS is known to be a substrate for both breast cancer resistance protein and MRP4 (Suzuki *et al*. 2003; Zelcer *et al*. 2003). In mouse brain, DHEAS uptake increased after systemic treatment with GF120918 (Lee *et al*. 2005), but similar results to ours were obtained by Bourasset *et al*. (2003), using the P‐gp specific inhibitor PSC833 (Boesch *et al*. 1991) in an attempt to inhibit the P‐gp mediated efflux of morphine‐6‐glucuronide, when brain uptake was decreased rather than increased by PSC833. Thus, the above three efflux inhibitors may not be as specific as previously thought, at least at the concentrations used, and may interact with other transport processes including uptake transporters; indeed PSC833 has been shown to interact with OATP2 *in vitro* (Cvetkovic *et al*. 1999). Finally, we cannot exclude the possibility that steroid sulphate transport at the abluminal membrane becomes limiting upon inhibition of steroid sulphatase and that GF120918 or MK571 are acting as inhibitors of this enzyme.

Addition of non‐radioactive substrate (0.5 mM) to the GF120918 and MK571 in the perfusion fluid resulted in a further reduction of the uptake for both radio‐labelled steroid sulphates, providing evidence for the involvement of an uptake mechanism. Asaba *et al*. (2000) have implicated OATP2 (now oatp1a4) in the uptake of DHEAS by rat brain but, as mentioned above, showed apparent efflux across the BBB to exceed this uptake. The efflux of DHEAS is impaired in oat3‐deficient but not oatp1a4‐deficient mice (Miyajima *et al*. 2011) and oat‐3 (organic anion transporter 3) is expressed at the abluminal membrane of the rat BBB (Mori *et al*. 2003). Involvement of anion transporters is further indicated by the attenuation of DHEAS brain uptake caused by the aryl sulfamate COUMATE given systemically in the mouse, even at doses which inhibit steroid sulphatase and increase the amount of DHEAS in circulation (Nicolas and Fry 2007). The OATPs (organic ion transporting polypeptides) and OATs (organic ion transporters) act as bidirectional transporters and may therefore be involved in uptake and/or efflux. New members of these families have recently been identified at the BBB (see Sugiyama *et al*. 2003; Tachikawa *et al*. 2014; Nigam *et al*. 2015) and an overlap of substrates and differences in expression between species are becoming apparent.

Further investigations are required to identify the transporters responsible for the influx and efflux of steroid sulphates across the BBB and to characterise their saturation kinetics. In this regard, the rapid and extensive (> 50%) desulphation of both PregS and DHEAS seen on crossing the BBB in the present study will presumably alter their concentration gradients in favour of influx. Moreover, the present results indicate that, even if there is a net efflux of the sulphated steroid, there will be an overall gain of free steroid into the brain. This interpretation is supported by the higher proportion of free than sulphated steroid accumulating in the brain parenchyma as opposed to the capillary fraction following perfusion through the carotid artery. This contrasts with results reported after intracerebral injection of DHEAS in rats, when Kishimoto and Hoshi (1972) found a higher proportion of sulphated than free steroid in blood plasma and Asaba *et al*. (2000) reported 90% of the ^3^H‐label appearing in the jugular vein after intracerebral ^3^H‐DHEAS to be the unchanged steroid sulphate. Thus, the free steroid liberated by deconjugation of the steroid sulphates upon uptake across the BBB is likely to be retained by the brain. There have been few reports of transport of PregS at the BBB, so the similarities that we have demonstrated between DHEAS and PregS are of interest. In humans, the most likely physiological conditions for net uptake of free steroid into brain are from DHEAS when this steroid reaches its highest plasma concentration in young adulthood (Orentreich *et al*. 1984) and from PregS (and 17α‐hydroxypregnenolone sulphate) during pregnancy (Vcelakova *et al*. 2007).

**Figure 6:**
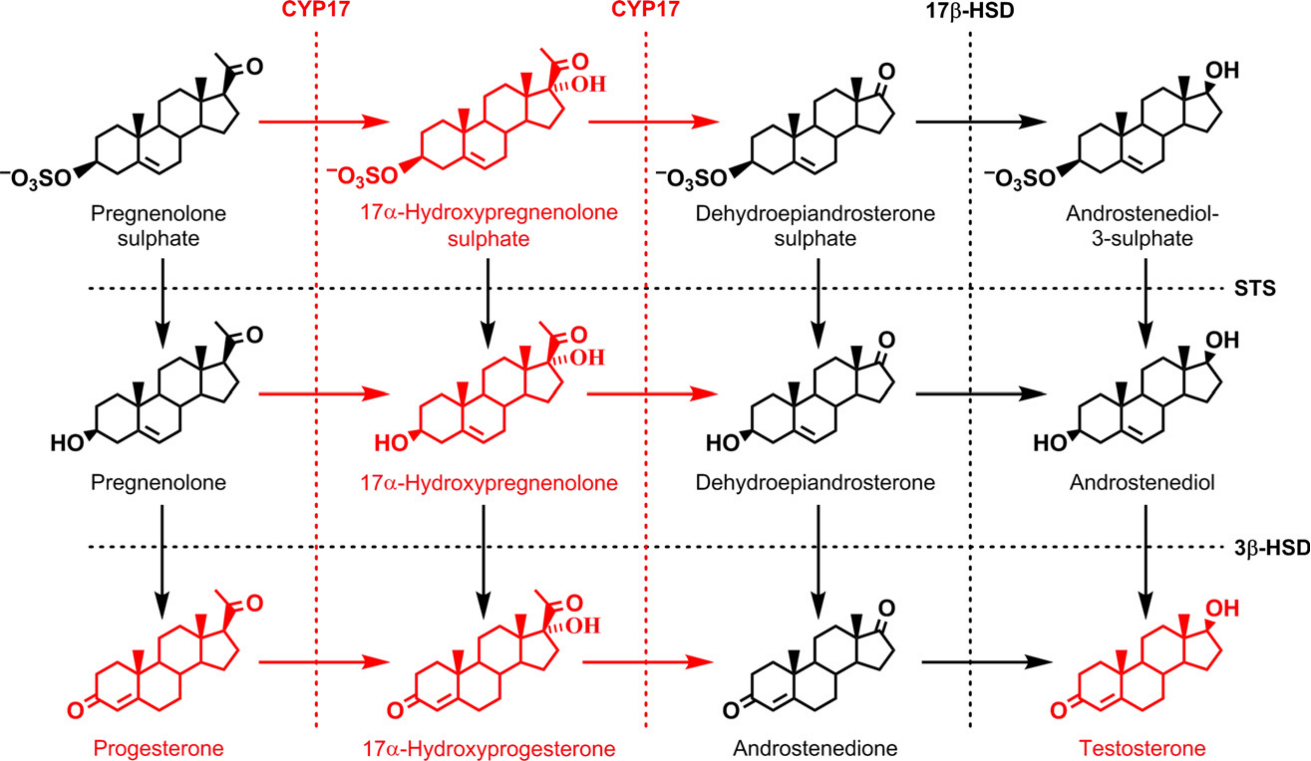
Pathways of pregnenolone sulphate and dehydroepiandrosterone sulphate metabolism after uptake into adult male rat brain. Metabolites identified in the present study are shown in black and those possible but not identified shown in red. Enzyme activity is indicated by dotted lines with the following abbreviations: CYP17, 17‐hydroxylase/c17,20‐lyase (EC 1.14.99.9); 3β‐HSD,3β‐hydroxy‐Δ^5^‐steroid:NAD ^+^ 3‐oxidoreductase (EC 1.1.1.145); 17β‐HSD, 17β‐hydroxysteroid:NAD(P)^+^ oxidoreductase (EC 1.1.1.51); STS, steroid sulphatase (EC 3.1.6.2). Enzymes previously identified by others are shown in black and, again, activity not found shown in red.

Consistent with the lack of CYP17 activity in the brain (see Introduction), the present investigation revealed no detectable conversion of either PregS or the deconjugated free steroid to the 17α‐hydroxy derivative or onwards to DHEA(S) (see Fig. 6), a major metabolic pathway in the periphery (Vcelakova *et al*. 2007). Previous analyses of endogenous brain steroids have also failed to detect the 17α‐hydroxypregnenolone intermediate which would be formed if CYP17 was catalysing the conversion of Preg to DHEA in this tissue (Ebner *et al*. 2006). Indeed, the free Preg generated from brain uptake of PregS did not undergo any detectable further metabolism during the 0.5 min perfusion of our experiments. However, enzymes which convert Preg to progesterone and further reduced metabolites are known to be active in rat brain (see Ebner *et al*. 2006) and presumably generate the increased brain progesterone, 5α‐dihydroprogesterone and allopregnanolone seen 20 min after intravenous infusion of PregS by Cheney *et al*. (1995). Allopregnanolone is a potent sedative steroid (see Melcangi and Panzica 2014) but varying amounts of the parent PregS in brain following systemic administration of this excitatory steroid sulphate (see Introduction) could account for its variable interactions with barbiturate‐induced anaesthesia (Majewska *et al*. 1989; Wang *et al*. 1997a).

Unlike PregS and as shown schematically in Fig 6, DHEAS underwent metabolism in brain both before and after desulphation during the 0.5 min perfusion of the present study. Thus, both DHEA and its 17β‐hydroxylated metabolite androstenediol could be identified in the free steroid and the steroid sulphate fractions following perfusion with DHEAS. Comparable results were obtained in rat brain by Kishimoto and Hoshi (1972) following intracardiac injection of DHEAS. Also, Kishimoto (1973) demonstrated a rat brain microsomal enzyme activity which could 17β‐hydroxylate both DHEA and DHEAS. The 17β‐hydroxysteroid:NAD(P)^+^ oxidoreductase (EC 1.1.1.51) which would catalyse this conversion appears to be localised in astrocytes in rat brain (Pelletier *et al*. 1995) and would account for the rapid formation of androstenediol sulphate as DHEAS is taken up at the BBB. The present study has also shown further metabolism of DHEA in the free steroid fraction to androstenedione. However, there was no evidence for production of 7α‐hydroxy DHEA, the major DHEA metabolite of rat brain microsomes *in vitro* (Akwa *et al*. 1992). Further studies are necessary to identify the metabolites of PregS and DHEAS following uptake at the BBB and their likely sites of action but, as mentioned above, enzymes exist in brain to convert free Preg to progesterone and sedative steroids. As for the DHEA, androstenediol and androstenedione formed from DHEAS, these are androgenic (Mo *et al*. 2006) and could also be further metabolised to estrogens. The latter conversion would require aromatase activity, which has been shown to enhance the neuroprotective effects of DHEAS in the rat (Juhasz‐Vedres *et al*. 2006). If at least part of the action of circulating DHEAS on the brain requires conversion to estrogens, then this may explain why its ability to enhance the acquisition and consolidation of memory in young laboratory animals is greater in males than in females, with their higher endogenous levels of estrogens (Sujkovic *et al*. 2012). Conversely, after the menopause in women, when ovarian secretion of estrogens has ceased and plasma DHEAS is declining with age more rapidly than in men (Orentreich *et al*. 1984), there is no gonadal source of androgens for conversion to estrogens and so females should be more sensitive to DHEA(S) supplementation than males (see Labrie 2010). This may account for the ability of short‐term (2 week) DHEA supplementation in elderly human subjects to improve mood and well‐being (but not cognitive function) in women rather than men (Wolf *et al*. 1997). Another possibility is for aromatase‐independent actions of DHEAS via androstenediol at estrogen receptor‐β (see Baker 2013; Warner and Gustafsson 2015). Whatever the central mechanism, the present results suggest that supplementation with DHEAS rather than DHEA may be a more effective way of boosting androgen concentrations in the ageing brain.

In their classic paper, Corpechot *et al*. (1981) suggested designation of DHEAS as a neurosteroid on the grounds that it persisted in adult male brain 15 days after adrenalectomy and castration, thereby indicating accumulation and/or synthesis within this tissue. Subsequent work (see Baulieu and Robel 1990; Asaba *et al*. 2000; Sujkovic *et al*. 2009; Miyajima *et al*. 2011) has argued against the accumulation of DHEAS because it is cleared from brain more rapidly than the 15 days of the Corpechot *et al*. (1981) study and yet, as summarised in the Introduction, the evidence remains equivocal for the synthesis of DHEA(S) within this tissue. The present results document a rapid uptake and desulphation of DHEAS at the BBB which accounts for the presence of this conjugate and its free steroid in adult rat brain. Our results are not inconsistent with those of Corpechot *et al*. (1981), who reported the concentration of DHEAS in the adult male rat plasma to remain unchanged 15 days after adrenalectomy and castration. Moreover, we have shown the desulphation of DHEAS and of PregS to be enriched in the BBB, which must play a key role in regulating not only the effects of these neuroactive steroids themselves but also their function as hormone precursors, giving rise to free steroids within the brain. Integrating the permeability and transport properties of the BBB with the metabolic pathways operating within the CNS gives a more complete understanding of the action of neuroactive steroids, building on the concept of the intracrinology of the protected CNS microenvironment beyond the BBB.

## Supporting information


**Figure S1.** Two examples of phosphor images from TLC plates showing the purity of ^3^H‐dehydroepiandrosterone (DHEAS) and of ^3^H‐pregnenolone sulphate (PregS).
**Figure S2.** Typical profiles of radioactivity (Intensity in arbitrary units (au)), following TLC of the steroid sulphate fraction from the parenchyma of rat brains perfused with (a) ^3^H‐dehydroepiandrosterone sulphate (^3^H‐DHEAS) or (b) ^3^H‐pregnenolone sulphate.
**Figure S3.** Typical profiles of radioactivity in arbitrary units (au) following TLC of (b) the desulphated steroid sulphate fraction and (c) the free steroid fraction from the parenchyma of rat brains perfused with ^3^H‐pregnenolone sulphate. Chromatography was in solvent system B and the positions of steroid standards indicated in panel (a) above the profiles.
**Figure S4.** Example profiles of radioactivity following TLC of the acetylated putative pregnenolone yielded by previous TLC (see Fig. S3) of either (b) the desulphated steroid sulphate fraction or (c) the free steroid fraction of the brain parenchyma of a rat perfused with ^3^H‐pregnenolone sulphate.
**Figure S5.** Typical profiles of radioactivity in arbitrary units (au) following TLC of (b) the desulphated steroid sulphate fraction and (c) the free steroid fraction from the parenchyma of rat brains perfused with ^3^H‐dehydroepiandrosterone sulphate (DHEAS)
**Figure S6.** Example profiles of radioactivity in arbitrary units (au) following TLC of the acetylated putative dehydroepiandrosterone (DHEA; b and d) or androstenediol (c and e) yielded by prior TLC (see Fig. S5) of either the desulphated steroid sulphate fraction (b and c) or the free steroid fraction (d and e) from the parenchyma of rat brains perfused with ^3^H‐DHEAS.
