# Supplementary material for: Uptake and metabolism of sulphated steroids by the blood–brain barrier in the adult male rat
Source: J Neurochem. 2017 Aug 3;142(5):672–85. doi: 10.1111/jnc.14117 (PMC5601180; doi:10.1111/jnc.14117)
Supplement: Supplementary file 1 — Figure S1. Two examples of phosphor images from TLC plates showing the purity of 3H‐dehydroepiandrosterone (DHEAS) and of 3H‐pregnenolone sulphate (PregS). Figure S2. Typical profiles of radioactivity (Intensity in arbitrary units (au)), following TLC of the steroid sulphate fraction from the parenchyma of rat brains perfused with (a) 3H‐dehydroepiandrosterone sulphate (3H‐DHEAS) or (b) 3H‐pregnenolone sulphate. Figure S3. Typical profiles of radioactivity in arbitrary units (au) following TLC of (b) the desulphated steroid sulphate fraction and (c) the free steroid fraction from the parenchyma of rat brains perfused with 3H‐pregnenolone sulphate. Chromatography was in solvent system B and the positions of steroid standards indicated in panel (a) above the profiles. Figure S4. Example profiles of radioactivity following TLC of the acetylated putative pregnenolone yielded by previous TLC (see Fig. S3) of either (b) the desulphated steroid sulphate fraction or (c) the free steroid fraction of the brain parenchyma of a rat perfused with 3H‐pregnenolone sulphate. Figure S5. Typical profiles of radioactivity in arbitrary units (au) following TLC of (b) the desulphated steroid sulphate fraction and (c) the free steroid fraction from the parenchyma of rat brains perfused with 3H‐dehydroepiandrosterone sulphate (DHEAS) Figure S6. Example profiles of radioactivity in arbitrary units (au) following TLC of the acetylated putative dehydroepiandrosterone (DHEA; b and d) or androstenediol (c and e) yielded by prior TLC (see Fig. S5) of either the desulphated steroid sulphate fraction (b and c) or the free steroid fraction (d and e) from the parenchyma of rat brains perfused with 3H‐DHEAS. [file JNC-142-672-s001.pdf]

Supporting Information

For

Uptake and metabolism of sulphated steroids by the blood–brain barrier in the adult male rat

By

M. Zeeshan Qaiser[*,1], Diana E. M. Dolman*, David J. Begley*, N. Joan Abbott*, Mihaela Cazacu-Davidescu†, Delia I. Corol†, Jonathan P. Fry†

*Blood–Brain Barrier Research Group, Institute of Pharmaceutical Science, Faculty of Life Sciences & Medicine, King's College London, London, SE1 9NH, UK and †Department of Neuroscience, Physiology and Pharmacology, University College London, Gower Street, London, WC1E 6BT, UK

[1]Present address: Pharmidex, 14 Hanover Street, London, W1S 1YH, UK

Address correspondence and reprint requests to Jonathan Fry, Department of Neuroscience, Physiology and Pharmacology, University College London, Gower Street, London, WC1E 6BT, UK

Tel: +44 (0) 7679 6214

E-mail: j.fry@ucl.ac.uk

Section 1. Example phosphor images from thin layer chromatography (TLC) plates showing purity of ^{3}H-dehydroepiandrosterone sulphate (DHEAS) and ^{3}H-pregnenolone sulphate (PregS) used in the present study.

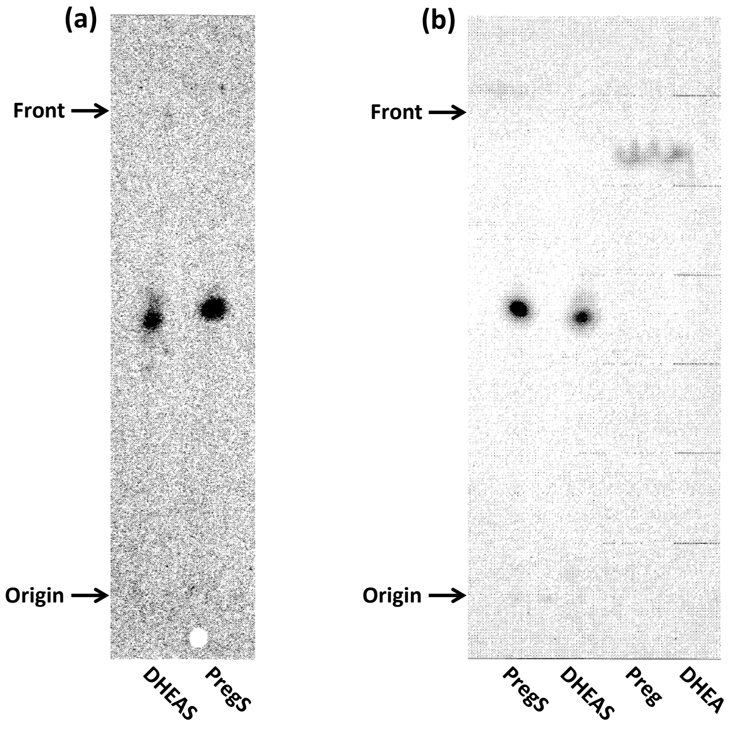

Fig. S1 Two examples of phosphor images from TLC plates showing the purity ^{3}H-dehydroepiandrosterone (DHEAS) and ^{3}H-pregnenolone sulphate (PregS). The white spot in (a) is from a hole punched for filing and (b) also shows the distribution of the free steroids ^{3}H-pregnenolone (Preg) and ^{3}H-dehydroepiandrosterone (DHEA). Both plates were run in ethyl acetate:ethanol:ammonia (25:10:2, v/v).

Section 2. Evidence that the labels ^{3}H-DHEAS, ^{3}H-DHEA, ^{3}H-PregS and ^{3}H-Preg are not altered during extraction and fractionation of steroids from the rat brain samples.

As shown in the legend to Fig. 4 of the main text, control experiments (*n* = 4) in which standard ^{3}H-DHEAS, ^{3}H-DHEA, ^{3}H-PregS or ^{3}H-Preg were added to rat brain homogenates followed by extraction and separation into free steroid and steroid sulphate fractions, as for the perfused rat brain samples, showed negligible sulphation or desulphation of the labels during these procedures. Thus the rapid desulphation of ^{3}H-DHEAS or ^{3}H-PregS seen on their uptake into the brain was not an artefact of the subsequent extraction and fractionation of steroids. Further TLC showed that there was no other detectable metabolism of these labels during the extraction and fractionation procedure. Thus, the steroid sulphates separated from homogenates labelled with ^{3}H-DHEAS or ^{3}H-PregS gave single peaks of radioactivity on TLC in solvent system A at Rf values of 0.51 ± 0.01 and 0.53 ± 0.01, respectively. Likewise, the free steroids separated from homogenates labelled with ^{3}H-DHEA and ^{3}H-Preg gave single peaks of radioactivity on TLC in solvent system B at Rf values of 0.35 ± 0.01 and 0.43 ± 0.01 respectively, and all the above peaks, corresponded to their appropriate ^{3}H-labelled standards.

Section 3. Distributions of radioactivity following TLC of steroid sulphate and free steroid fractions from the brain parenchyma of rats perfused with either ^{3}H-DHEAS or ^{3}H-PregS.

Positions of these steroids on TLC are given below relative to the solvent front (Rf) as mean ± SEM. Typical profiles of radioactivity as detected by phosphorimaging are also shown, with intensity in arbitrary units (au). The positions of standards are indicated by horizontal bars above the profiles. Non-radioactive steroid standards were visualised by exposure to iodine vapour whereas the ^{3}H-steroids were detected by the phosphorimager.

TLC of steroid sulphate fractions from the parenchyma of ^{3}H-DHEAS-perfused and from ^{3}H-PregS-perfused rat brains

Upon TLC in solvent system A, the steroid sulphate fractions from the parenchyma of ^{3}H-DHEAS-perfused (n = 5) and from ^{3}H-PregS-perfused (n = 5) rat brains gave single peaks corresponding to their appropriate standards, although that from the ^{3}H-DHEAS-perfused rats formed a wider peak (Rf 0.45 ± 0.01 to 0.60 ± 0.01) than the ^{3}H-DHEAS standard (Rf 0.53 to 0.64; see Fig. S2).

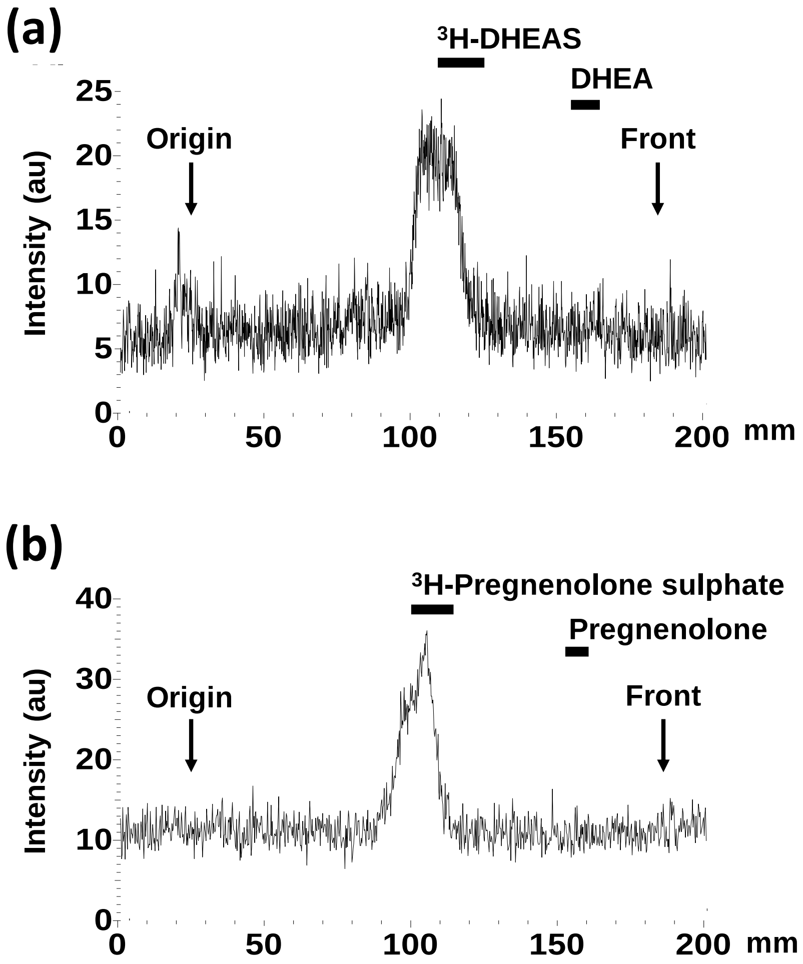

Fig. S2 Typical profiles of radioactivity (Intensity in arbitrary units (au)), following TLC of the steroid sulphate fraction from the parenchyma of rat brains perfused with (a) ^{3}H-dehydroepiandrosterone sulphate (^{3}H-DHEAS) or (b) ^{3}H-pregnenolone sulphate. Chromatography was in solvent system A and the positions of steroid standards are indicated by horizontal bars above the profiles.

TLC of the desulphated steroid sulphate fraction from the parenchyma of ^{3}H-PregS-perfused rat brains in comparison with the free steroid fraction from the same parenchyma samples

Upon TLC in solvent system B, both the ^{3}H-label from the steroid sulphate fractions and the standard ^{3}H-PregS gave peaks on desulphation corresponding (Rf = 0.45 ± 0.01) to standard Preg (Rf = 0.44). Likewise, TLC of the free steroid fraction from these ^{3}H-PregS-perfused rat brains in the same solvent system showed no evidence of metabolism other than desulphation, with peaks (Rf = 0.45 ± 0.01) corresponding to standard Preg and not to other possible Preg metabolites (see Fig. S3).

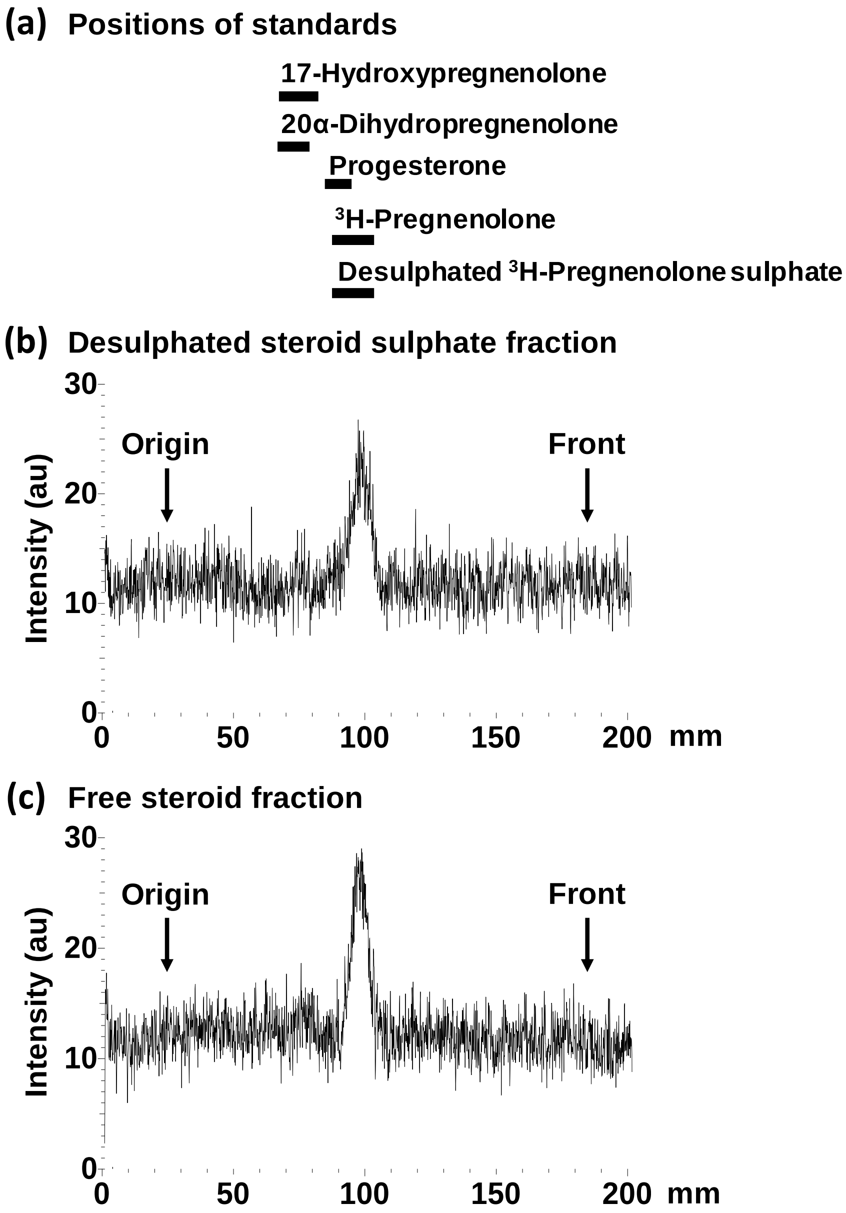

Fig. S3 Typical profiles of radioactivity in arbitrary units (au) following TLC of (b) the desulphated steroid sulphate fraction and (c) the free steroid fraction from the parenchyma of rat brains perfused with ^{3}H-pregnenolone sulphate. Chromatography was in solvent system B and the positions of steroid standards indicated in panel (a) above the profiles.

TLC of the putative Preg isolated from the desulphated steroid sulphate fraction and the free steroid fraction from the parenchyma of ^{3}H-PregS-perfused rat brains, following acetylation alongside known steroid standards

Upon TLC in solvent system C, both standard ^{3}H-Preg and standard desulphated ^{3}H-PregS gave two peaks after acetylation: at Rf = 0.26 and 0.41. These two peaks corresponded with those from the acetylated putative Preg peaks of the free steroid fractions at 0.26 ± 0.01 and 0.41± 0.01 and of the desulphated steroid sulphate fractions at 0.26 ± 0.01 and 0.41 ± 0.01 (see Fig. S4). These two peaks arise from a presumed Serini reaction in which the Preg forms isomeric enol acetates (Fieser & Huang-Minlon, 1949, J Am Chem Soc, 71, 1840-1842). Acetylated non-radioactive Preg carried through the above procedure gave only one stain with iodine at Rf = 0.42, probably representing the less stable trans isomer.

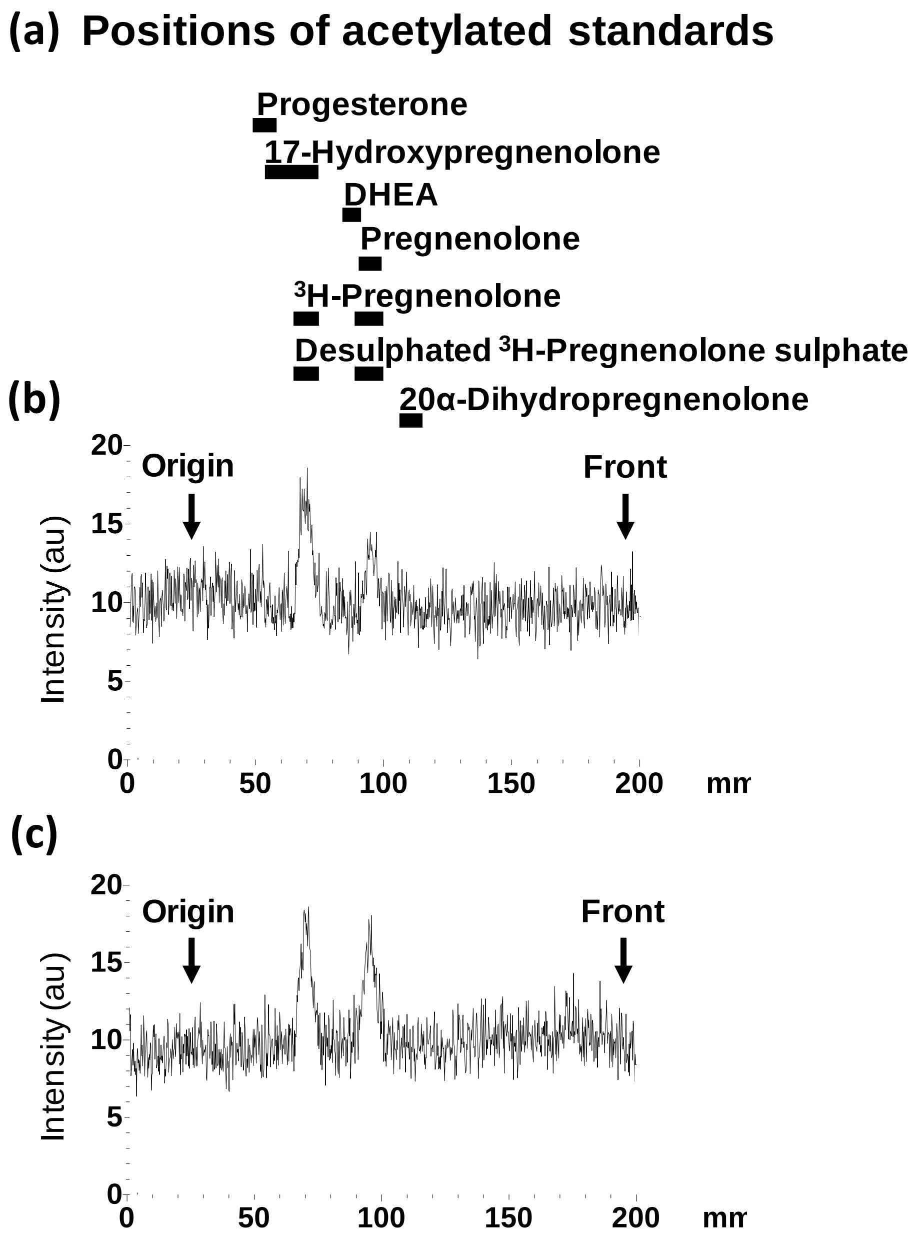

Fig. S4 Example profiles of radioactivity following TLC of the acetylated putative pregnenolone yielded by previous TLC (see Fig. S3) of either (b) the desulphated steroid sulphate fraction or (c) the free steroid fraction of the brain parenchyma of a rat perfused with ^{3}H-pregnenolone sulphate. Chromatography was in solvent system C and positions of acetylated standards are shown in panel (a) above the profiles.

TLC of the desulphated steroid sulphate fraction from the parenchyma of ^{3}H-DHEAS-perfused rat brains in comparison with the free steroid fraction from the same parenchyma samples

Upon TLC in solvent system B, both the desulphated steroid sulphate fraction and the free steroid fraction from the parenchyma of ^{3}H-DHEAS-perfused rat brains gave two peaks: from the desulphated steroid sulphate fraction at Rf 0.29 ± 0.01 and Rf 0.38 ± 0.01 and from the free steroid fraction at Rf 0.28 ± 0.01 and Rf 0.38 ± 0.01 (see Fig. S5). The earlier peaks from both fractions corresponded with androstenediol (Rf = 0.25) and the later peaks with ^{3}H-labelled and non-radioactive DHEA at Rf = 0.36. There were no detectable 7-hydroxymetabolites of DHEA (Rf < 0.05). The two peaks which arose from the steroid sulphate fraction were not an artefact of the deconjugation procedure because desulphation of the ^{3}H-DHEAS standard gave only one peak at 0.37.

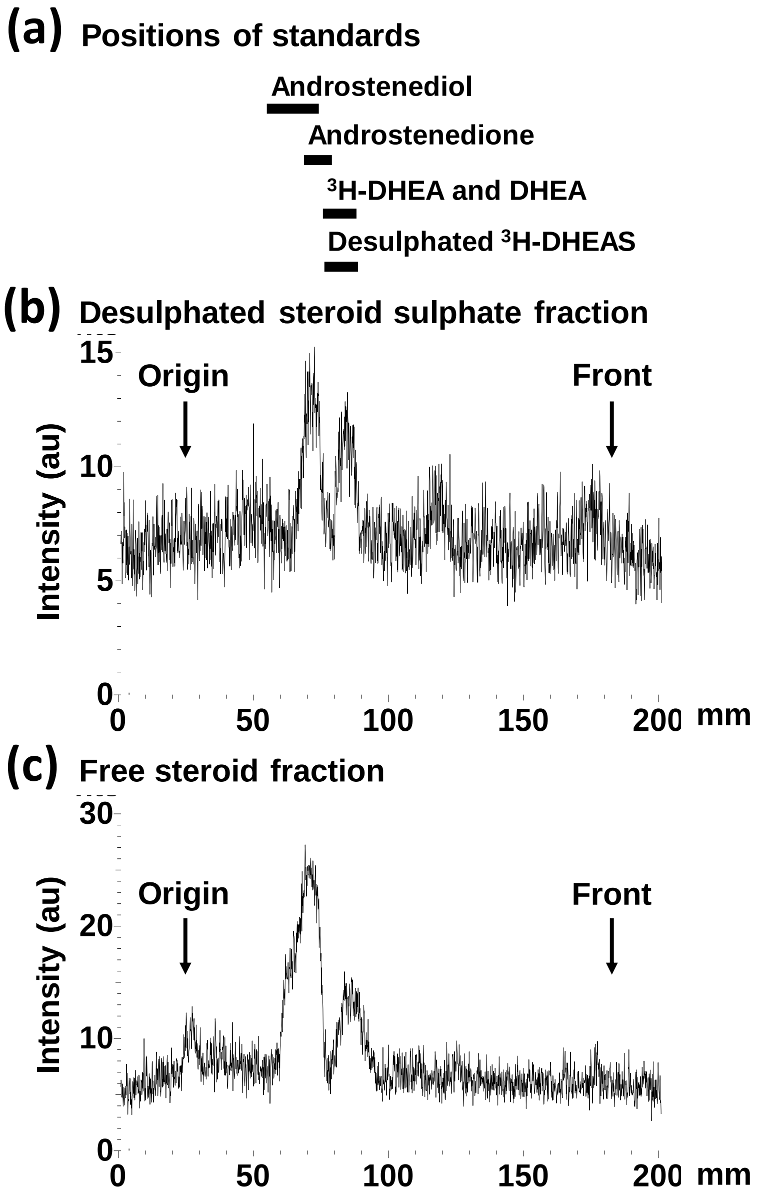

Fig. S5 Typical profiles of radioactivity in arbitrary units (au) following TLC of (b) the desulphated steroid sulphate fraction and (c) the free steroid fraction from the parenchyma of rat brains perfused with ^{3}H-dehydroepiandrosterone sulphate (DHEAS). Chromatography was in solvent system B and the positions of steroid standards indicated in panel (a) above the profiles.

TLC of the putative DHEA and androstenediol isolated from the desulphated steroid sulphate fraction and the free steroid fraction from the parenchyma of ^{3}H-DHEAS-perfused rat brains, following acetylation alongside known steroid standards

Following acetylation and TLC in solvent system C, the putative DHEA from both the desulphated steroid sulphate fractions and the free steroid fractions gave single peaks at Rf = 0.38 ± 0.01 and Rf = 0.36 ± 0.01, respectively. These peaks corresponded with acetylated standard ^{3}H-labelled (Rf = 0.38) and non-radioactive DHEA (Rf = 0.37). Likewise, acetylation of the putative androstenediol gave peaks from the desulphated steroid sulphate fractions at Rf = 0.47 ± 0.01 and from the free steroid fractions at 0.45 ± 0.01, both corresponding with acetylated standard androstenediol at Rf = 0.46 (see Fig. S6). However, acetylation of the ^{3}H-label eluted from the putative androstenediol peak in the free steroid fraction also gave two additional peaks, one of which Rf = 0.11 ± 0.01 corresponded to standard androstenedione carried through the acetylation procedure (Rf = 0.14; although this would not be acetylated) and another Rf = 0.34 ± 0.01 which could not be identified. There was no ^{3}H-peak corresponding with standard acetylated testosterone (Rf = 0.24).

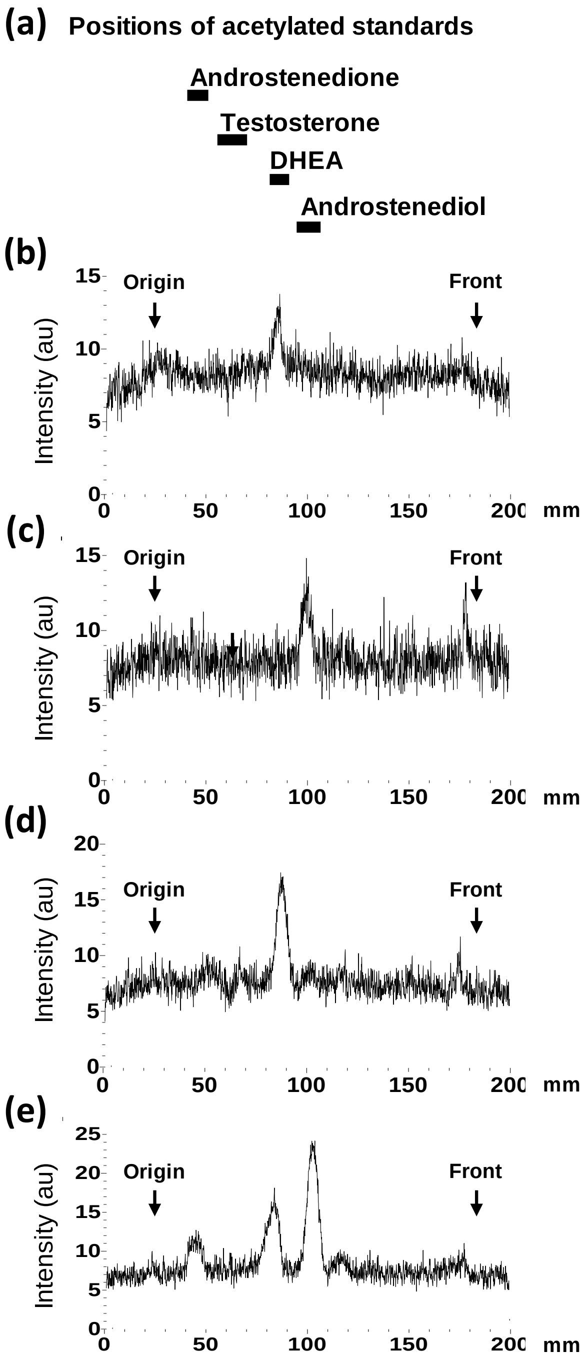

Fig. S6 Example profiles of radioactivity in arbitrary units (au) following TLC of the acetylated putative dehydroepiandrosterone (DHEA; b and d) or androstenediol (c and e) yielded by prior TLC (see Fig. S5) of either the desulphated steroid sulphate fraction (b and c) or the free steroid fraction (d and e) from the parenchyma of rat brains perfused with ^{3}H-DHEAS. Chromatography was in solvent system C and positions of acetylated standards are shown in panel (a) above the profiles.
